# Estrogenic microenvironment generated by organochlorine residues in adipose mammary tissue modulates biomarker expression in ERα-positive breast carcinomas

**DOI:** 10.1186/bcr1534

**Published:** 2006-07-19

**Authors:** Mónica Muñoz-de-Toro, Milena Durando, Pablo M Beldoménico, Horacio R Beldoménico, Laura Kass, Silvia R García, Enrique H Luque

**Affiliations:** 1 Laboratorio de Endocrinología y Tumores Hormonodependientes, School of Biochemistry and Biological Sciences, Universidad Nacional del Litoral, Santa Fe, Argentina; 2 Faculty of Sciences, University of Liverpool, UK; 3 Central Laboratory, Pesticide Division, School of Chemical Engineering, Universidad Nacional del Litoral, Santa Fe, Argentina

## Abstract

**Introduction:**

Breast cancer is the most frequent malignant disease in women. Exposure to estrogens throughout a woman's life is a risk factor for the development of breast cancer. Organochlorine compounds (OCCs), such as pesticides and polychlorinated biphenyls, are persistent lipophilic chemicals identified as endocrine disruptors, mainly with estrogenic effects. To test the hypothesis that the amount and quality of organochlorine residues in adipose tissue adjacent to breast carcinoma affect the biological behavior of the tumor, we studied biomarker expression in breast carcinoma and the OCC body burden in patients from an urban area adjacent to Paraná fluvial system, Argentina.

**Methods:**

The studied patients were 55 women who had undergone excision biopsies of a breast lesion diagnosed as invasive breast carcinoma. Analysis of OCC residues in breast adipose tissue was conducted by electron-capture gas–liquid chromatography. Estrogen receptor alpha (ERα), progesterone receptor (PR) and proliferative activity (Ki-67) levels were measured in paraffin-embedded biopsies of breast tumors by immunohistochemistry.

**Results:**

All patients had high levels of organochlorine pesticides in their breast adipose tissue. The most frequently detected compounds were *p*,*p'*-dichlorodiphenyldichloroethylene, hexachlorobenzene and β-hexachlorocyclohexane. When the whole sample was analyzed, no correlation between ERα or PR expression and OCC levels were found. In the subgroup of ERα-positive breast carcinoma patients, however, there was a positive correlation between PR expression (an estrogen-induced protein) in the neoplastic cells and OCC levels in adipose tissue surrounding the tumor. More significantly, all the ERα-positive breast carcinomas from postmenopausal women exhibited high proliferation when organochlorine levels in the surrounding adipose tissue reached levels higher than 2600 ppb. No associations were found between the organochlorine body burden and any other marker of tumor aggressiveness, such as node involvement or tumor size.

**Conclusion:**

The present results support the hypothesis that organochlorine residues in adipose tissue adjacent to breast carcinoma generate an estrogenic microenvironment that may influence the biological behavior of the tumor through ERα activation and ERα-dependent proliferation. These findings may have therapeutic implications, since interference between organochlorine compounds and hormonal therapy could be expected to occur.

## Introduction

Breast cancer is the most frequent malignant disease in women. Exposure to estrogens throughout a woman's life, including the period of intrauterine development, is a risk factor for the development of breast cancer [1-3]. Epidemiological studies suggest a strong correlation between estrogen and/or xenoestrogen exposure and cancer, which is supported by numerous *in vivo *and *in vitro *experimental studies that attest to the proliferative and carcinogenic potency of estrogens [4].

Organochlorines present in the environment include agricultural pesticides and industrial compounds. Chemically stable and strongly lipophilic, organochlorine compounds (OCCs) have slow degradation rates and tend to bioaccumulate in lipid-rich tissues. Because some OCCs act as estrogen agonists they have been linked to an increased incidence of breast cancer [5,6], although not all data have been consistent [7-9].

People are exposed to OCCs from the environment and from residues in food. We recently reported that women from Santa Fe City area, a littoral region in Argentina, had high levels of organochlorine pesticides in their breast adipose tissue, and, more significantly, *p*,*p'*-dichlorodiphenyldichloroethylene (*p*,*p'*-DDE) was present in all sampled women and at high concentrations. The diet was a major factor that influenced the organochlorine pesticide body burden. Fish, particularly fatty varieties caught in local rivers, as well as other fat-rich meats were significant sources of exposure [10].

It is of interest, alongside designing studies to establish a link between xenoestrogens and breast cancer risk, to determine whether or not changes in the tumor microenvironment, due to organochlorine accumulation and release, affect the biological behavior of the tumor. In a nested case–control study, it has recently been demonstrated that patients with increased numbers of epithelial cells expressing estrogen receptor alpha (ERα) in normal lobular units are at increased risk of subsequent invasive breast cancer [11]. These results suggest that overexpression of estrogen receptor in benign breast epithelium may increase estrogen sensitivity, thereby creating a permissive state that can lead to malignancy. As ERα mediates mammary cell differentiation and function, inappropriate exposure of cells to estrogens or xenoestrogens might alter the behavior of an established neoplasia. This might have therapeutic implications [12]. Contradictory results have been reported in studies that addressed the possible relationship between organochlorine levels, breast cancer aggressiveness and prognosis [13-16]. These differences may be due to methodological approaches since organochlorine levels were measured in different physiological compartments (adipose tissue and serum samples), which results in inappropriate comparisons [17,18].

We hypothesized that the amount and quality of the organochlorine residues in adipose tissue adjacent to breast carcinoma might affect the biological behavior of the tumor. To test this hypothesis we conducted a cross-sectional study evaluating whether or not the amount and/or quality of organochlorine residues adjacent to breast carcinomas are associated with the expression of biological markers (paying special attention to hormone-dependent and hormone-independent breast cancer markers). We studied a population of women with breast lesions who reside in Santa Fe City and nearby locations, an urban area on the coast of Paraná River in a region that is characterized by intensive agricultural activities. This agricultural activity favors an elevated prevalence of environmental exposures, which our laboratory has recently confirmed [10].

## Materials and methods

### Study subjects

Participants included in this report were a subsample of subjects who were enrolled in a study to assess the OCC body burden among residents of Santa Fe City area in Argentina [10]. This subsample (*n *= 55) comprised women that underwent an excision biopsy of a breast lesion diagnosed as invasive breast carcinoma between March 1998 and April 1999 in regional hospitals of Santa Fe City. All patients were urban residents at the time of surgery, were nonoccupationally exposed to persistent OCCs and were subjected to no therapeutic intervention before biopsy. An informed consent form was signed by all participants, 49 of whom completed a questionnaire.

The questionnaire provided information about sociodemographic factors, menopausal status, weight loss, height, parity, breast-feeding, dietary habits and hormone replacement therapy. Since OCC residue removal during lipolysis could be expected, periods of breast-feeding and weight loss before surgery were included in the questionnaire. The interview was conducted by a trained social worker by telephone and/or home visit. Histopathological reports and medical records were available for all patients. The primary tumor extent (T1–T4) and lymph node status were determined using the American Joint Committee on Cancer criteria [19]. Approval for this study was obtained from the Universidad Nacional del Litoral Institutional Review Board (Santa Fe, Argentina).

### Tissue sample collection

At the time of biopsy 0.5–1.0 g breast adipose tissue adjacent to the lesion was collected and weighed, was frozen at -70°C and was stored until organochlorine analysis. Paraffin blocks of breast tumor samples, previously fixed in 10% buffered formalin for 24 hours at room temperature, were available for immunohistochemical evaluation of tumor biomarkers.

### Laboratory assays

#### Detection of organochlorine in adipose tissue

Breast adipose tissue was analyzed for the presence of the following compounds: hexachlorobenzene, hexachlorocyclohexane isomers (α-hexachlorocyclohexane, β-hexachlorocyclohexane and lindane), aldrin, oxychlordane, α-chlordane, γ-chlordane, heptachlor, heptachlor epoxide, dieldrin, endrin, mirex, metoxychlor, DDE (*p*,*p'*-DDE and *o*,*p'*-DDE), tetrachlorodiphenylethane (*p*,*p'*-tetrachlorodiphenylethane, *o*,*p'*-tetrachlorodiphenylethane), dichlorodiphenyltrichloroethane(*p*,*p'*-DDT, *o*,*p'*-DDT) and eight polychlorinated biphenyl congeners (International Union of Pure and Applied Chemistry numbers 077, 118, 128, 138, 170, 180, 187 and 195).

Concentrations of organochlorines were measured following the method previously described by Muñoz-de-Toro and colleagues [10]. Briefly, an aliquot of 500 mg adipose tissue was dehydrated and then extracted in acetone–petroleum ether (25:75, v/v). The extract was filtered and the container rinsed with petroleum ether. All the petroleum ether fractions were collected and evaporated to dryness, and the percentage of fat in each tissue sample was calculated. Then, an aliquot of 200 mg fat from each sample was dissolved in 5 ml petroleum ether. Solid-phase extraction cleanup was performed using Supelclean LC-Alumina-N SPE tubes (Supelco, Bellefonte, PA, USA), and a second cleanup was performed using Supelclean Envi-Florisil SPE tubes (Supelco). Ten milliliters of ethylic ether–petroleum ether (15:85, v/v) was used to elute the chlorinated compounds from the microcartridge.

A gas–liquid chromatograph (Varian™ model 3400, Varian Inc., Palo Alto, CA, USA) equipped with a ^63^Ni electron-capture detector was used for the analysis of residues. The gas chromatography (GC) columns used were: a Megabore Capillary GC Column DB 608 (30 m × 0.53 mm ID, film thickness 0.83 μm; J&W Scientific, Taejon, Korea), a GC Capillary Column CP-Sil 8 CB (50 m × 0.25 mm ID, film thickness 0.12 μm; VWR International B.V., Amsterdam, The Netherlands), and a GC packed column (2 m × 2 mm) coated with 1.5% SP 2250 + 1.95% SP 2401 on a Supelcoport 100–120 ID mesh (VWR International B.V., West Chester, PA, USA).

Organochlorine pesticide standards Pestanal (Honeywell Riedel-de Haën Fine Chemicals, Seelze, Germany) and polychlorinated biphenyl congener standards (Ultra Scientific, North Kingstown, RI, USA) were used. All the solvents used were of pesticide-grade quality (Merck, Darmstadt, Germany).

The OCC presence and level were confirmed using a GC–mass spectrometry system (VG Trio 2; VG Analytical, Manchester, UK) in randomly selected samples. The operational quality control and limits of detection have been reported previously [10].

#### Tumor biomarkers

ERα expression, progesterone receptor (PR) expression and Ki-67 expression were measured by immunohistochemistry as previously described [20]. Samples received microwave pretreatment for antigen retrieval. Primary antibodies used were ERα (clone 6F-11) and PR (clone 1A6) from Novocastra Laboratories (Newcastle upon Tyne, UK) and Ki-67 (clone MIB-1; Amac, Westbrook, ME, USA), diluted 1:60, 1:20 and 1:100, respectively. Incubation with primary antibody was performed at 4°C for between 14 and 16 hours. Biotinylated anti-mouse IgG (Sigma, St Louis, MO, USA) was used as a second antibody; biotin was detected with the streptavidin–peroxidase (Sigma) complex. Diaminobenzidine tetrahydrochloride (Sigma) was used as a chromogen.

Biomarker expression was measured in the epithelial compartment. A proliferation index was obtained by considering the percentage of all Ki-67-positive cells independently of staining intensity (2,000 cells were counted/tissue section and at least three sections per sample were included). The Ki-67 assay (MIB-1) values were categorized according to Trihia and colleagues [21] as follows: low proliferation, ≤ 9.5%; intermediate proliferation, >9.5 and ≤ 15.5%; and high proliferation, >15.5%. ERα and PR were quantified by considering the percentage of positive cells, and the results were categorized into three groups [22]: negative, 0%; weakly positive, >0 and <10%; and strongly positive, ≥ 10%.

### Data analysis

The variables evaluated were organochlorine concentrations, age at biopsy (years), menopause status at biopsy (pre/post), age at menopause (years), years between menopause and surgery, years lived in a rural area, occurrence of weight loss (>3 kg) during the year before biopsy (yes/no), body mass index (kg/m^2^), number of children, months of lactation (averaged when several lactations and categorized into three groups: less than 3 months; from 3 to 6 months; and more than 6 months), animal fat intake (categorized into two groups: low, patients who ate lean red meat and processed meat (sausage, salami, etc.) infrequently; and high, those who ate red meat and processed meat regularly), freshwater fish intake (less than once a week, or at least once a week), and dairy product intake (low, infrequent; intermediate, once a week; or high, every day). Each food type was evaluated individually and then all food types were evaluated together as a score summation.

In addition, the variables tumor size (categorized as T1, 0–2 cm; 2 < T2 < 5 cm; T3, ≥ 5 cm; and T4, any size that invades muscle or skin), lymph node metastasis (none or present), and ERα, PR and MIB-1 expression (percentages and categories, described earlier) were evaluated. Expressions of ERα and PR were also dichotomized as positive and negative to conduct stratified analysis.

Nondetectable levels of OCCs were estimated as the mean between 0 and the detection limit. The OCC values were log-transformed when parametric tests were used. The central tendency of organochlorine residues was reported as geometric means.

All possible pairs of variables were tested for associations by appropriate statistical tests. Parametric tests were used when the assumptions were completely met, and nonparametric tests were used when assumptions were not met (Mann–Whitney tests to compare two groups, Kruskal–Wallis tests to compare three or more groups of a nominal variable, Jonckheere–Terpstra tests to compare three or more groups of an ordinal variable, and Spearman's Rho correlation to compare two continuous variables). Pearson and Mantel–Haenszel chi-square tests and Fisher's exact tests were used to compare proportions.

Results are reported for the pairs that were of interest (i.e. organochlorine residues and tumor biomarker expression), whereas the other pairs were used to assess the probability that an association of interest was caused by confounding or interaction effects of a third variable. Although multivariable analysis was not possible due to the limited sample size, stratification was conducted every time it was of interest to assess the associations within values of a third variable.

## Results

### Patient and tumor characteristics

Table 1 presents summary descriptive information on the study subjects. All tumors were classified as invasive carcinomas, and the tumor size varied widely (T1 = 30.2%, T2 = 52.8%, T3 = 5.7%, and T4 = 11.3% of our breast carcinoma patients). Node-positive patients (one or more axillary lymph nodes with neoplastic cells) comprised 46.9% of participants.

Data on tumor biomarker expression are summarized in Table 2. According to hormone receptor expression, 50.0% of carcinomas appeared to be hormone-dependent tumors (ERα-positive and PR-positive) and 30.8% were hormone-independent tumors (ERα-negative and PR-negative). Breast carcinomas from premenopausal patients showed a lower percentage of ERα-positive cells (12.2 ± 19%) than those from postmenopausal patients (42.3 ± 35.5%; Mann–Whitney test, *P* = 0.021). ERα expression in breast tumors from node-positive patients (23.3 ± 32.4%) appeared to be lower than in tumors from node-negative women (43.6 ± 34.2%), but this difference was not statistically significant (Mann–Whitney test, *P *= 0.065). PR-negative breast carcinomas showed a higher proliferative index (33.4 ± 15.7%) than PR-positive carcinomas (22.0 ± 11.5%; Mann–Whitney test, *P *= 0.015).

There was a positive association between animal fat intake and tumor proliferative index (Fisher's exact test, *P *= 0.025): 69.7% of tumors with high proliferation were from patients with high fat consumptions. On the other hand, dairy product intake was positively correlated with ERα expression in carcinomas: 0%, 23.8% and 42.1% for low consumption, intermediate consumption and high consumption, respectively (Jonckheere–Terpstra test, *P *= 0.017).

### Organochlorine levels in breast adipose tissue

A considerable high frequency of occurrence of organochlorine residues and elevated concentrations of organochlorine residues were found in breast adipose tissue of all participants (*n *= 55) (Table 3). The residues found most frequently were *p*,*p'*-DDE in all patients analyzed, hexachlorobenzene in 87.3%, β-hexachlorocyclohexane in 74.5%, heptachlor epoxide in 36.4%, oxychlordane in 32.7%, and heptachlor in 21.8%. *p*,*p'*-DDT was detected in three patients (5.5%). The concentrations of organochlorine residues in breast adipose tissue were very high, with maximum values of 4794 ppb for *p*,*p'*-DDE and 1780 ppb for β-hexachlorocyclohexane.

### Organochlorine levels and characteristics of the adjacent tumors

Analysis of all subjects found no associations between the organochlorine body burden and any marker of tumor aggressiveness such as node involvement, tumor size or proliferative activity. Moreover, the 12 patients who died within 4 years following surgery had organochlorine levels that did not differ from those of the surviving studied subjects (Mann–Whitney test, *P *= 0.303).

**Figure 1:**
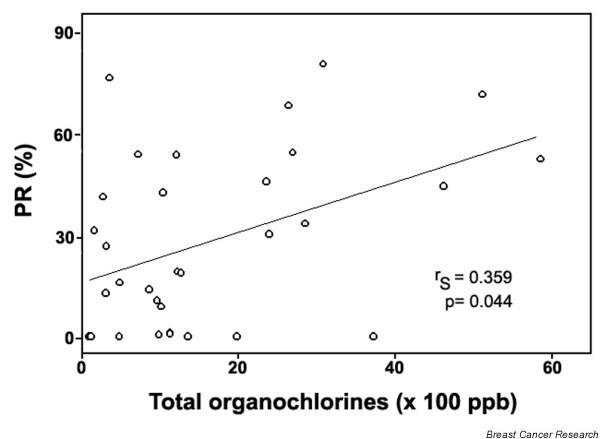
**Organochlorine levels modulate progesterone receptor expression in estrogen receptor-α positive breast carcinomas**. The percentage of PR expression in ERα-positive breast carcinoma was positively correlated with organochlorine levels in the adjacent adipose tissue (Spearman's Rho test, *P *= 0.044).

Regarding levels of organochlorine and the expression of biomarkers in adjacent breast tumors, concentrations of organochlorine appeared higher when surrounding hormone-dependent (ERα-positive and PR-positive) tumors (geometric means for total organochlorines in hormone-dependent tumors = 1169.2 ng/g versus hormone-independent tumors = 711.1 ng/g); however, this trend was not statistically significant (Mann–Whitney test, *P *= 0.379). Interestingly, in the subgroup of ERα-positive tumors, a significant positive correlation was observed between PR expression and total organochlorine levels (Spearman's Rho correlation, *P *= 0.044) (Figure 1), mainly due to *p*,*p'*-DDE levels since the strongest correlation was observed between PR expression and *p*,*p'*-DDE (Spearman's Rho correlation, *P *= 0.010).

**Figure 2:**
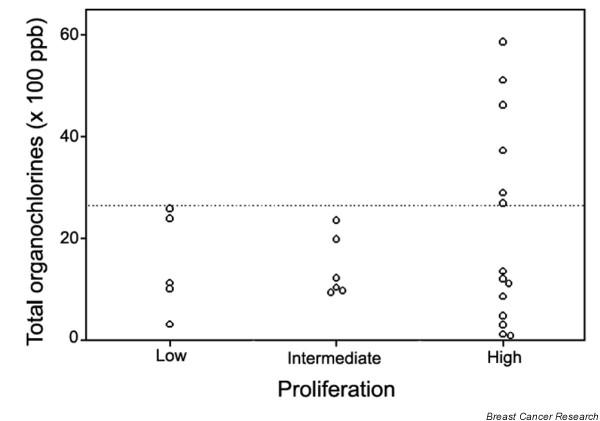
**High organochlorine levels promote high proliferation in estrogen receptor-α positive breast carcinomas of postmenopausal**. All ERα-positive breast carcinomas from postmenopausal women had high cellular proliferation when levels of organochlorine residues were 2600 ppb or higher (above the dashed line) (Fisher's exact test, *P *= 0.017 – statistical analysis of tumors with low and intermediate proliferation grouped).

The estrogenic effect of OCCs was also evident when tumor proliferative activity was evaluated in ERα-positive breast tumors from postmenopausal women. As shown in the subgroup of ERα-positive breast tumors from postmenopausal patients (Figure 2), all the adjacent tumors had high cellular proliferation when levels of OCC residues were 2600 ppb or higher (above the dashed line) (Fisher exact test, *P *= 0.017 – statistical analyses of tumors with low and intermediate proliferation were grouped). Furthermore, it is interesting to note the bimodal distribution of the OCC concentrations in the group of highly proliferative ERα-positive breast carcinomas from postmenopausal patients (Figure 2). The subgroup clustered above the 2600 ppb threshold were patients that had been menopausal for a long time (mean 31 years), while those with low total OCCs (<2600 ppb) and high proliferation had been in menopause for a shorter time (mean 17 years; Mann–Whitney test, *P *= 0.032). Moreover, when ERα-positive tumors from premenopausal patients were analyzed, all tumors with high proliferation had low total OCC levels (data not shown).

In other words, patients that had been menopausal for a long time exhibited high proliferation if OCC levels surrounding the tumor were high, whereas highly proliferative ERα-positive breast carcinomas with a microenvironment of low OCC levels were seen either in premenopausal women or in women that went through menopause more recently.

## Discussion

This study presents evidence regarding an estrogenic effect of OCCs on human mammary tumors that developed in a rich OCC environment. The PR expression in ERα-positive breast carcinomas was positively associated with OCC concentration in the adjacent adipose tissue, while in the subgroup of postmenopausal women, when low serum levels of endogenous estrogen are expected, high levels of OCCs in the adipose tissue surrounding breast tumor were associated with high proliferative activity. The present results support the hypothesis that organochlorine residues in adipose tissue adjacent to breast carcinoma generate an estrogenic microenvironment that may influence the biological behavior of the tumor.

We recently demonstrated that women living in an urban area of an agricultural and industrial region of Argentina had high levels of organochlorine residues in their breast adipose tissue [10]. More significantly, *p*,*p'*-DDE was present in all subjects at high concentrations. In the present report, the study subjects were a subsample of women from our previous work [10] who were diagnosed with invasive breast carcinoma. As expected, these women also exhibited high levels of organochlorine residues in the adipose tissue adjacent to the breast tumors.

Epidemiologic studies have identified numerous prognostic parameters in breast cancer patients. Among these parameters, tumor size, cell proliferation, lymph node involvement and steroid receptor status are accepted as the most significant [23]. Even though our sample size is small, our results agree with randomized clinical studies performed to provide prognostic information in patients with breast carcinoma [23]. We have found that premenopausal and node-positive women showed a lower expression of ERα in their tumors, and that PR-negative carcinomas have a higher proliferative index. These observations agree with large well-designed clinical studies [23] and attest to the representativeness of our sample.

The relation of diet, especially fat intake, to recognized breast cancer prognostic indicators was investigated in humans as well in animal models. In rats, a high-fat diet significantly stimulated tumor proliferation and cell kinetics in chemically induced mammary carcinomas [24], while in women an increase in saturated fat intake was positively associated with tumor growth and/or spread [25]. Consistent with this, our tumors from patients that reported high fat consumption exhibited high proliferative indices. In the present study, dairy product intake was positively correlated with tumor ERα expression. This result suggests that these dietary components could modify the tumor biological behavior, improving the response to endocrine therapy and/or survival in breast carcinoma patients.

Previous studies on estrogenic organochlorine levels and breast cancer have focused mainly on risk assessment while few of them have taken into account hormone receptor status and/or biomarkers of tumor aggressiveness. Dewailly and colleagues [26] and Liljegren and colleagues [27] found that patients with ERα-positive tumors had higher DDE and a higher polychlorinated biphenyl body burden than controls, but Muscat and colleagues [16] failed to confirm this finding. In the present study, a trend of higher concentrations of total organochlorines surrounding hormone-dependent (ERα-positive and PR-positive) tumors was apparent; this trend was not statistically significant, possibly due to the small sample size. Most organochlorine pesticides found in our samples were defined as xenoestrogens [28]; consistent with this, a positive association was established in the ERα-positive breast tumor subgroup between PR expression and OCC levels in adjacent adipose tissue. This result suggests an upregulation of PR expression via an ERα-dependent mechanism.

DDT isomers and metabolites bind to and transcriptionally activate human ERα [29]. Moreover, organochlorine has been shown estrogenic in MCF-7 cells by the expression of PR [29]. Using a transgenic mouse model, Villa and colleagues [30] showed that PR mRNA was significantly induced by DDT isomers in tissues positive for estrogen receptors, and that the estrogenic action of *p*,*p'*-DDE was more efficient in organs that expressed ERβ, such as the mammary gland. Organochlorines might induce the observed changes in PR expression either directly by binding ERs or indirectly by increasing sensitivity to endogenous estrogens, or affecting the amount and type of estrogen metabolites with estrogenic properties. The local estrogen synthesis in neoplastic breast tissue depends on two principal pathways: the 'aromatase pathway', which transforms androgens into estrogens; and the 'sulfatase pathway', which converts estrone sulfate into estrone [31,32]. It is tempting to suggest that OCC could increase the local biosynthesis of estrogens through the modulation of the aforementioned enzymatic pathways.

Regarding the organochlorine body burden and the prognosis of breast carcinoma patients, adverse effects have been suggested by Demers and colleagues [13], Hoyer and colleagues [14] and Muscat and colleagues [16]. In the present study, the markers of aggressiveness such as tumor size or node involvement show no associations with the organochlorine body burden, while the proliferative activity was associated with OCC levels when low endogenous estrogen serum levels are expected (postmenopause). An elevated cellular proliferation in breast carcinoma patients that have been menopausal for a long time depended on the levels of OCCs, whereas high cellular proliferation in those premenopausal patients or those that had more recently gone through menopause appeared to be independent of OCC concentrations. Our result agrees with results recently obtained *in vitro*, where proliferation of MCF-7 was induced by xenoestrogens when tumor cells were cultured in a medium devoid of estrogen [33]. Our study has the characteristic that breast tumors in our series were diagnosed later than in developed countries, where active breast cancer screening programs work properly. Based on this observation, it is suggested that tumors in our series developed and progressed in an organochlorine-rich environment; following their natural history without intervention, thus providing more accurate information regarding the OCC effect on breast tumor biological behavior.

The present results highlight the importance of differentiating between tumor types, in terms of the estrogen receptor status and PR status, when analyzing the effects of both estrogens and xenoestrogens. The emerging picture is that epithelium and stromal compartments interact via a complex network of diffusible factors, resulting in the modulation of cell growth and differentiation, which facilitates tumor development and/or progression in a permissive, xenoestrogen-rich microenvironment. It is not known how the induction of cell proliferation and the increased expression of PR in tumors developed in an organochlorine-rich microenvironment would affect disease-related survival, as hormone-receptor-positive tumors may be associated with a more favorable prognosis compared with hormone-receptor-negative tumors [34].

In summary, the present results support the hypothesis that the amount and quality of the organochlorine residues in adipose tissue adjacent to breast carcinoma is responsible for generating an estrogenic microenvironment that may influence the tumor biological behavior. In the ERα-positive breast carcinoma, organochlorine levels in adipose tissue were associated with an increase in PR expression and cell proliferation, both mechanisms induced through estrogen action and ERα activation. While several *in vitro *works have shown the xenoestrogen effect on breast tumor cell lines [28,29,33], present results suggest the occurrence of *in vivo *estrogenic effects of organochlorine residues on human breast tumors. Also, the results suggest that tumors of women at advanced menopause depend upon a microenvironment of high xenoestrogen levels to induce high cell proliferation. New studies with larger samples would be necessary to confirm this preliminary result.

## Conclusion

The present results support the hypothesis that organochlorine residues in adipose tissue adjacent to breast carcinoma generate an estrogenic microenvironment that may influence the biological behavior of the tumor through ERα activation and ERα-dependent proliferation. These findings may have therapeutic implications, since interference between organochlorine compounds and hormonal therapy could be expected to occur.

## Abbreviations

DDE = dichlorodiphenyldichloroethylene; DDT = dichlorodiphenyltrichloroethane; ERα = estrogen receptor alpha; GC = gas chromatography; OCC = organochlorine compound; PR = progesterone receptor; T1–T4 = tumor size.

## Competing interests

The authors declare that they have no competing interests.

## Authors' contributions

MM-d-T participated in designing the study, acquiring data from medical records and histopathological reports, analyzing and interpreting data, and outlining and reviewing the manuscript. MD participated in designing the study, searching the literature, acquiring data (inmunohistochemistry), analyzing data and drafting the manuscript. PMB participated in designing the study, analyzing data (statistical analysis) and reviewing the manuscript. HRB participated in designing the study, and analyzing and interpreting data from electron-capture gas–liquid chromatography. LK participated in designing the study, searching the literature, acquiring data (inmunohistochemistry), analyzing data and reviewing the manuscript. SRG carried out the gas–liquid chromatography, and analyzed and interpreted data. EHL participated in designing the study, acquiring study samples, analyzing and interpreting data, and preparing the manuscript. All authors read and approved the final manuscript.

## Figures and Tables

**Table 1:** Characteristics of patients studied

**Continuous variables**	
Age (years)	60.5 ± 11. 6 (*n *= 51)
Body mass index (kg/m^2^)	25.4 ± 4.8 (*n *= 46)
Number of full-term pregnancies	2.2 ± 1.9 (*n *= 48)
Rural residence (years)	10.4 ± 17.8 (*n *= 45)
**Categorical variables**	
Breastfeeding (*n *= 45)	
<3 months total	35.6
>3 and <6 months total	11.1
>6 months total	53.3
Menopausal status (*n *= 49)	
Premenopausal	24.5
Postmenopausal	75.5
Weight loss (>3 kg) during the year before biopsy (*n *= 42)	
No	76.2
Yes	23.8
Freshwater fish consumption (*n *= 47)	
Less than once a week	68.1
At least once a week	31.9
Dairy product intake (*n *= 46)	
Infrequent	6.5
At least once a week	17.4
Every day	76.1
Animal fat intake (*n *= 46)	
Low	34.8
High	65.2

Continuous variables presented as the mean ± standard deviation; categorical variables presented as the percentage.

**Table 2:** Biomarkers of hormone dependence and proliferative activity in breast carcinomas

Immunophenotype^a^	Percentage of cases (*n*)
Hormone dependence	
ERα-positive/PR-positive	50.0 (26)
ERα-positive/PR-negative	11.5 (6)
ERα-negative/PR-positive	7.7 (4)
ERα-negative/PR-negative	30.8 (16)
MIB-1 category	
Low proliferation	12.8 (6)
Intermediate proliferation	17.0 (8)
High proliferation	70.2 (33)

^a^Criteria adopted to establish cut-off values are described in Materials and methods. ERα, estrogen receptor alpha; PR, progesterone receptor.

**Table 3:** Concentrations of organochlorine residues in breast adipose tissue adjacent to a malignant lesion

Compound	Frequency (%)	Geometric mean (95% confidence interval)	Range (ppb)
*p*,*p'*-Dichlorodiphenyldichloroethylene	100	541.9 (398.3–737.3)	20–4,794
Hexachlorobenzene	87.3	33.9 (22.2–51.6)	BDL-252
β-Hexachlorocyclohexane	74.5	81.7 (43.1–154.8)	BDL-1,780
Heptachlor epoxide	36.4	5.5 (3.8–7.9)	BDL-222.0
Oxychlordane	32.7	6.5 (4.0–10.5)	BDL-622.0
Heptachlor	21.8	2.8 (2.3–3.4)	BDL-24
*p*,*p'*-Dichlorodiphenyltrichloroethane	5.5	2.5 (1.9–3.1)	BDL-159
*o*,*p'*-Dichlorodiphenyldichloroethylene	1.8	2.2 (1.8–2.6)	BDL-255
Dieldrin	1.8	2.1 (1.9–2.3)	BDL-34
Total polychlorinated biphenyls	1.8	2.1 (1.9–2.4)	BDL-81
Total organochlorines	100	885.3 (646.6–1,212.1)	20.0–5,861

BDL, below detection limit.
